# Crowd-sourcing and author submission as alternatives to professional curation

**DOI:** 10.1093/database/baw149

**Published:** 2016-12-26

**Authors:** Peter D. Karp

**Affiliations:** Bioinformatics Research Group, SRI International, 333 Ravenswood Ave, Menlo Park, CA 94025, USA. Tel:650-859-4358; Fax: 650-859-3735; e-mail: pkarp@ai.sri.com

## Abstract

Can we decrease the costs of database curation by crowd-sourcing curation work or by offloading curation to publication authors? This perspective considers the significant experience accumulated by the bioinformatics community with these two alternatives to professional curation in the last 20 years; that experience should be carefully considered when formulating new strategies for biological databases. The vast weight of empirical evidence to date suggests that crowd-sourced curation is not a successful model for biological databases. Multiple approaches to crowd-sourced curation have been attempted by multiple groups, and extremely low participation rates by ‘the crowd’ are the overwhelming outcome. The author-curation model shows more promise for boosting curator efficiency. However, its limitations include that the quality of author-submitted annotations is uncertain, the response rate is low (but significant), and to date author curation has involved relatively simple forms of annotation involving one or a few types of data. Furthermore, shifting curation to authors may simply redistribute costs rather than decreasing costs; author curation may in fact increase costs because of the overhead involved in having every curating author learn what professional curators know: curation conventions, curation software and curation procedures.

## Introduction

Can we decrease the costs of database curation by crowd-sourcing curation work or by offloading curation to publication authors? Bourne et al. proposed (1) these two alternatives to professional curation. Both alternatives are forms of manual curation by individuals who are not primarily trained and paid to be professional curators. The bioinformatics community has accumulated significant experience with these two alternatives to professional curation in the last 20 years; that experience should be carefully considered when formulating new strategies for biological databases, such as the funding cuts being considered for several model-organism databases (2, 3) and the funding cuts recently applied by NIH to the EcoCyc and MetaCyc databases (The NIH grant for EcoCyc was cut by 18% at its last renewal; the NIH grant supporting MetaCyc/BioCyc was cut by 27% at its last renewal.).

## Crowd sourcing as an alternative to professional curation

Even before the founding of Wikipedia in 2001, crowd-sourcing of curation (also known as community curation), was an appealing possibility for the life sciences. Community annotation was envisioned as a major advantage of The Genome Sequence Database (GSDB) (4) compared with Genbank. GSDB developed a community annotation tool called GSDB Annotator to facilitate annotation contributions from scientists. However, few community-contributed annotations were received by GSDB, which was among the reasons for its demise several years later.

Dr M. Cherry reports that another experiment in community curation was performed by the Saccharomyces Genome Database (SGD). After receiving encouragement from the yeast community at an experimental conference, SGD authored a software tool for web-based submission of community curation. So little curation was submitted by the yeast community that this tool was discontinued by SGD.

The EcoCyc and MetaCyc projects also experimented with a type of community curation in the 2000s. Aware of past low participation rates for community curation, and postulating that reviewing existing database entries would take less time than authoring new entries, we began systematically contacting authors of articles we had curated to request that they review the gene annotations and pathways we had curated. Again, the response rate was so low that we discontinued the practice.

More recently, the EcoliWiki project asked *Escherichia coli* scientists to contribute information on *E. coli* genes, proteins, and strains. Despite engaging and persistent advocacy by the project’s director, Dr J. Hu, at multiple *E. coli* conferences, the response rate was fairly low (data supplied by Hu show that from 2007 to 2010, an average of 46 people per year contributed 583 wiki page updates; but from 2013 to 2015 the numbers decreased to an average of 10 people per year contributing 76 updates).

The Gene Wiki project (5) has created Wikipedia pages for all human genes that combine data that was programmatically extracted from structured databases with text authored by unpaid human contributors. These articles contain 1.42 million words of text contributed by 6830 distinct editors for >10 000 genes. However, since the information within Gene Wiki articles are not captured within a structured ontology, these Wiki data are not accessible to computational analysis, thus the successes of the Gene Wiki project are not easily transferable to curation of structured databases. Similar comments apply to the Rfam database (6) and its use of Wikipedia to curate textual descriptions of RNA families.

## Direct curation by authors

Author-submitted curation is a type of crowd-sourced curation in which the curator is the person who knows the published work best and will benefit the most from the promotion of the work. Several moderate success stories are emerging for author-submitted curation.

The TAIR project allows authors of submitted articles to submit Gene Ontology (GO) term annotations on *Arabidopsis* genes at the time they submit an article. Dr T. Berardini, who supervises TAIR curation, states that TAIR has received 800 such submissions over the years; in 2015, 87 authors submitted 2686 GO annotations for 98 articles. TAIR director Dr E. Huala believes that the reasons for this success include that TAIR has established relationships with plant journals that ask authors to submit data; that data are submitted at the time authors are most excited about publicizing their work; because the online submission form is simple; and that authors realized that curating their articles in TAIR raises the profile of their work in the scientific community.

Canto is a web-based tool designed to allow publication authors to enter biological knowledge about genes, proteins and protein interactions (7). Canto was developed by PomBase for fission yeast literature curation but was designed to be easily deployed for other organisms, or to use additional ontologies. Canto provides a series of web-based forms that allow an author curator to specify what genes are mentioned in an article, and to specify GO terms, protein modifications, interactions, phenotypes, and alleles for those genes. Overall, authors have submitted 5300 distinct annotations from 300 publications to PomBase via Canto (8). In 2015, 18% of annotations entering PomBase were submitted by authors via Canto, and 82% were entered by professional curators (8), meaning that author curation made a significant dent in the curation workload.

FlyBase speeds publication triage by sending email requests to authors of newly published articles requesting that, via an online tool, the authors list the genes studied in a publication and indicate the types of data described in the article (9). The author response rate to the FlyBase email requests was a respectable 44% over a nine-month period. Bunt et al. found that author response rates to these email requests were higher for recently published articles than if authors were contacted 2–13 months after publication of their article (35% response rate). On a yearly basis, Bunt et al. report that this author triage system frees up 2–3 months of FlyBase curator time per year that would have otherwise been spent on article triage.

## Discussion

It is interesting that all of the success stories come from the author-curation model rather than the crowd-sourced curation model. We should note that since authors are members of ‘the crowd’, nothing would prevent authors of an article from participating in crowd-sourced curation. However, it appears that explicitly targeting authors to participate, particularly near the time of publication when they are most enthusiastic about the work, yields a significantly higher success rate than wider appeals to ‘the crowd’.

But let us consider other issues and trade-offs around the author-curation model.

Bourne et al. are certainly concerned with the costs of biological databases, and particularly with curation costs. But will shifting work from professional curators to the crowd or to authors really save money? Probably some members of the crowd work for free, such as retired scientists or hobbyists. Yet crowd-sourced curation appears to have a very low participation rate, so its cost-saving potential seems quite low.

In addition, some members of the crowd will seek payment for their work, and authors are usually professional (paid) scientists, so to a degree I see a shell game here—costs are simply being shifted from one bin (professional curators) to another (authors). That is, whether a curator is being paid for N hours of work or an author is being paid for M hours of work, someone is still being paid. We can argue about who works more efficiently, or about the notion that if the authors who do the curation are graduate students who make lower salaries than professional curators, or work from other funding sources than government grants, the NIH may save some money here. The point is that author time costs money too, and every hour an author spends curating is taking away from their time in the laboratory.

But one could also argue that professional curators who are more familiar than authors with curation practices and curation software will also curate faster and more accurately than authors. Have Bourne et al. identified a key inefficiency in their statement ‘There is an unnecessary cost in a researcher interpreting data and putting that interpretation into a research article, only to have a biocurator extract that information from the article and associate it back with the data’? Indeed, why not have the people who understand the work best—the authors—enter their results directly into one or more relevant databases? One reason is that professional curators have unique skills and training that the average bench scientist lacks. Indeed, in our 20+ years of experience in developing curated databases, we have found that some PhD-level life scientists cannot develop into successful curators even after prolonged training.

Curator training encompasses multiple topics. One topic is curation conventions, since a significant goal of biological databases is to standardize the inconsistent terminology found in the life-sciences literature. For example, EcoCyc defines conventions for naming proteins, metabolites and metabolic pathways. The EcoCyc Curator’s Guide (10) also defines conventions for defining the boundaries of metabolic pathways, conventions for what units to use for different database fields, style guidelines for writing mini-review summaries for genes and pathways, citation guidelines and conventions for assigning evidence codes to database entries.

Curators also receive training in how to use the curation software used to enter new information into a database. For example, in EcoCyc, we enter a new metabolic pathway by first entering each metabolic reaction in the pathway (which involves entering reactant and product compounds not already present in the database), and then defining the pathway itself. Separate editing tools exist for metabolites, for reactions, for enzymes and for pathways. Each is fairly complicated to use, for example, the reaction editor includes a reaction-balance checker and allows users to specify reaction-directionality information as well as the cellular compartment(s) in which a reaction occurs. This software is nearly impossible to use without significant study or training. Curators are also trained in the methods needed to ensure that the information that is entered into a database is amenable to computational analysis, such as the use of ontologies, and the persistent determination to refrain from entering stray commentary and other non-conformant text into controlled database fields. Ultimately these methods ensure that the EcoCyc database can be computationally converted into an executable metabolic model, thus avoiding the need (and cost) of having separate curation efforts for a model-organism database and a metabolic model that now occur for most organisms.

For review-level databases it may be preferable that synthesis of information from multiple articles be performed by neutral third parties such as database curators. Another unappreciated role of professional curators is to correct the errors that are rampant throughout the experimental literature; if database entries resulting from a publication were authored by the same person who authored the publication, they would likely promulgate the same errors from the publication into the database; fresh eyes are more likely to notice errors.

In my view the inefficiency identified by Bourne et al. of having a person different from the author curate an article is more than offset by multiple inefficiencies of the author-curates model where one author of every curated publication must learn curation methods, conventions, ontologies and software—probably for multiple databases over the course of multiple publications! Given the lack of interest most scientists have shown in crowd-sourced curation, it seems likely that if curation were forced upon them, some authors would take shortcuts in the process, skimping on what information they enter, and circumventing curation methods. The result will be incomplete, low-quality database entries.

As discussed in (11), there is variation in the complexity of different curation tasks. We posit that ‘the ease of replacing professional curators with some other approach (here, author curation) will depend on the complexity of the curation to be performed’*.* For example, it will be more challenging for authors to curate multiple types of data (e.g. gene functions, gene-regulation mechanisms and sequence variation) than a single type of data (e.g. gene functions alone).

If database budgets are slashed by funding agencies, will scientists come to the rescue, such as by volunteering their time to assist in database curation? Many databases have lost their funding over the years; I know of no instance where scientists have come to the rescue in this way. For example, for many years the National Science Foundation biological databases program had a policy of funding databases for one grant cycle only; few if any of the databases funded under this program found any alternative source of funding after the first cycle. We do have the recent example of the TAIR Arabidopsis database, which lost its funding a few years ago and has now begun a successful subscription model to raise funds for curation and operations from the scientific community (12). In this case scientists came to the aid of a database by purchasing subscriptions.

## Conclusions

The vast weight of empirical evidence to date suggests that crowd-sourced curation is not a successful model for biological databases. Multiple approaches to crowd-sourced curation have been attempted by multiple groups, and extremely low participation rates by “the crowd” are the overwhelming outcome.

The author-curation model developed by TAIR, Canto, and FlyBase does show promise for boosting curator efficiency, and should be explored by other databases. However, note its limitations. This model has taken years to develop. The quality of author-submitted annotations is still uncertain (and should not be taken for granted given the complexity of GO). The response rate is significant but low. And to date author curation has involved relatively simple forms of annotation involving one or a few types of data. Furthermore, shifting curation to authors may simply redistribute costs rather than decrease costs; author curation may in fact increase costs because of the overhead involved in having every curating author learn what professional curators know: curation conventions (e.g. naming and style guidelines), curation software, and curation procedures.

The more complex the database, the more the balance is likely to tip in favor of professional curation because authors will require more training to produce the high-quality curation achieved by professional curators.

## Funding

This work was supported by Award Number GM077678 from the National Institute of General Medical Sciences of the National Institutes of Health. The content of this article is solely the responsibility of the authors and does not necessarily represent the official views of the National Institutes of Health.

*Conflict of interest*. None declared.
